# A Gluten Reduction Is the Patients’ Choice for a Dietary ‘Bottom Up’ Approach in IBS—A Comment on “A 5Ad Dietary Protocol for Functional Bowel Disorders” *Nutrients* 2019, *11*, 1938

**DOI:** 10.3390/nu12010137

**Published:** 2020-01-03

**Authors:** Christian Charles Shaw, Rachel Louise Buckle, Anupam Rej, Nick Trott, Imran Aziz, David Surendran Sanders

**Affiliations:** 1 Academic Unit of Gastroenterology, Royal Hallamshire Hospital, Sheffield Teaching Hospital NHS Foundation Trust, Sheffield S10 2JF, UK; rachel.buckle2@nhs.net (R.L.B.); anupam.rej@nhs.net (A.R.); nick.trott@nhs.net (N.T.); imran.aziz1@nhs.net (I.A.); david.sanders1@nhs.net (D.S.S.); 2 Academic Unit of Gastroenterology, Department of Infection, Immunity and Cardiovascular Disease, University of Sheffield, Sheffield S10 2JF, UK

**Keywords:** low FODMAP diet, gluten free diet, irritable bowel syndrome (IBS)

We read the article by Ibrahim and Stribling [1] with interest, as dietary therapies for functional disorders can be challenging to implement, such as the initial phase of the low fermentable oligo-, di-, mono- saccharide and polyols (FODMAPs) diet. In view of this, streamlined approaches to achieve symptom management maybe warranted, such as a ‘bottom up’ approach. Such therapies remove over-restriction and potentially have fewer concerns regarding nutritional adequacy [2].

The authors voice concerns regarding the effectiveness of dietary therapies for functional bowel disorders. However, several studies demonstrate the growing evidence for the use of traditional dietary advice, low FODMAP diet (LFD) and gluten free diet (GFD) for Irritable bowel syndrome (IBS) [3].

We would disagree that the 5Ad protocol is a ‘bottom up’ approach, but rather a ‘top down’ approach, as it involves restriction of high FODMAPs and other dietary components. In contrast, a GFD could be viewed as a ‘bottom up’ approach, through the reduction of fructans, with a response rate of 30–71% to this diet [3]. Recent research demonstrated patients use gluten free products, classed as ‘low fructans foods’, whilst on the LFD personalisation stage [4].

There is limited evidence for the 5Ad protocol and it remains unknown to the public, unlike the GFD [5]. In addition, the GFD has been rated as more acceptable than the LFD [6]. Only 40% of individuals have been shown to follow the LFD correctly [7], highlighting the need for easier approaches such as a gluten restriction.

We further disagree with the authors criticism that the LFD and GFD do not support healthy eating. The authors refer to the LFD in its entirety as the restriction stage, failing to acknowledge the further two LFD stages, which aim to ensure nutritional adequacy [8]. O’Keeffe and Colleagues (2018) [4]. demonstrated that nutritional adequacy was not compromised at long-term in the LFD group or in those returning to a habitual diet 6–18 months follow up.

The nutritional adequacy of the 5Ad remains to be explored, as nutritional analysis was performed on a ‘model’ diet, shedding no light on how the diet may actually be followed by participants, or if it was during the intervention.

The 5Ad diet is vague on the reintroduction of foods, providing no food quantity guidance and concerningly, does not emphasise the importance of food reintroduction. In contrast, a GFD can be viewed as less restrictive than the 5Ad diet, as well as the LFD, as there is no requirement for food reintroduction.

In conclusion, the 5Ad may be viewed as a restrictive dietary approach, with the efficacy and nutritional adequacy of this diet yet to be determined. Less restrictive options such as a gluten restriction as a ‘bottom up’ approach to reduce fructans, is biologically feasible, and should be an option for patients.

## References

[1] Ibrahim F., Stribling P. (2019). A 5Ad Dietary Protocol for Functional Bowel Disorders. Nutrients.

[2] Wang X.J., Camilleri M., Vanner S., Tuck C. (2019). Review article: Biological mechanisms for symptom causation by individual FODMAP subgroups—The case for a more personalised approach to dietary restriction. Aliment. Pharmacol. Ther..

[3] Rej A., Aziz I., Tornblom H., Sanders D.S., Simrén M. (2019). The role of diet in irritable bowel syndrome: Implications for dietary advice. J. Intern. Med..

[4] O’keeffe M., Jansen C., Martin L., Williams W., Seamark L., Staudacher H.M., Irving P.M., Whelan K., Lomer M.C. (2018). Long-term impact of the low-FODMAP diet on gastrointestinal symptoms, dietary intake, patient acceptability, and healthcare utilization in irritable bowel syndrome. Neurogastroenterol. Motil..

[5] Croall I.D., Trott N., Rej A., Aziz I., O’Brien D.J., George H.A., Hossain M.Y., Marks L.J.S., Richardson J.I., Rigby R. (2019). A population survey of dietary attitudes towards Gluten. Nutrients.

[6] Paduano D., Cingolani A., Tanda E., Usai P. (2019). Effect of three diets (low- FODMAP, gluten-free and balanced) on irritable bowel syndrome symptoms and health-related quality of life. Nutrients.

[7] Tuck C.J., Reed D.E., Muir J.G., Vanner S.J. (2019). Implementation of the low FODMAP diet in functional gastrointestinal symptoms: A real—World experience. Neurogastroenterol. Motil..

[8] Whelan K., Martin L.D., Staudacher H.M., Lomer M.C.E. (2018). The low FODMAP diet in the management of irritable bowel syndrome: An evidence-based review of FODMAP restriction, reintroduction and personalisation in clinical practice. J. Hum. Nutr. Diet..

